# Prevalence of *Shigella boydii* in Bangladesh: Isolation and Characterization of a Rare Phage MK-13 That Can Robustly Identify Shigellosis Caused by *Shigella boydii* Type 1

**DOI:** 10.3389/fmicb.2019.02461

**Published:** 2019-11-07

**Authors:** Mahmuda Akter, Nathan Brown, Martha Clokie, Mahmuda Yeasmin, Tokee M. Tareq, Ramani Baddam, Muhammad A. K. Azad, Amar N. Ghosh, Niyaz Ahmed, Kaisar A. Talukder

**Affiliations:** ^1^International Centre for Diarrhoeal Disease Research, Bangladesh (icddr,b), Dhaka, Bangladesh; ^2^Department of Genetics and Genome Biology, University of Leicester, Leicester, United Kingdom; ^3^Department of Biochemistry and Molecular Biology, Shahjalal University of Science and Technology, Sylhet, Bangladesh; ^4^Division of Electron Microscopy, ICMR-National Institute of Cholera and Enteric Diseases, Kolkata, India; ^5^Department of Biotechnology and Genetic Engineering, Mawlana Bhashani Science and Technology University, Tangail, Bangladesh

**Keywords:** shigellosis, *Shigella boydii* type 1, phage, diagnosis, low-cost

## Abstract

Shigellosis, caused by *Shigella boydii* type 1, is understudied and underreported. For 3 years, GEMS study identified 5.4% of all *Shigella* as *S. boydii*. We showed the prevalent serotypes of *S. boydii* in Bangladesh and phage-based diagnosis of *S. boydii* type 1, a rapid and low-cost approach. Previously typed 793 clinical *S. boydii* strains were used for serotype distribution. Twenty-eight environmental water samples were collected for isolation of *Shigella* phages. Forty-eight serotypes of *Shigella* and other enteric bacteria were used for testing the susceptibility to phage MK-13. Electron microscopy, restriction enzyme analysis, whole genome sequencing (WGS), and annotation were performed for extensive characterization. *S. boydii* type 1 is the second most prevalent serotype among 20 serotypes of *S. boydii* in Bangladesh. We isolated a novel phage, MK-13, which specifically lyses *S. boydii* type 1, but doesn’t lyse other 47 serotypes of *Shigella* or other enteric bacteria tested. The phage belongs to the *Myoviridae* family and distinct from other phages indicated by electron microscopy and restriction enzyme analysis, respectively. MK-13 genome consists of 158 kbp of circularly permuted double-stranded DNA with G + C content of 49.45%, and encodes 211 open reading frames including four tRNA-coding regions. The genome has 98% identity with previously reported phage, ΦSboM-AG3, reported to have a broader host range infecting most of the *S. boydii* and other species of *Shigella* tested. To our knowledge, MK-13 is the first phage reported to be used as a diagnostic marker to detect *S. boydii* type 1, especially in remote settings with limited laboratory infrastructure.

## Introduction

Shigellosis is an important cause of morbidity and mortality among preschool-aged and older children, and adults (Mani et al., 2016). Two recent studies, MAL-ED and GEMS, conducted in Bangladesh and other countries, identified *Shigella* as one of the four leading pathogens (Kotloff, 2017; Kotloff et al., 2017). GEMS identified *Shigella* and pathogenic *Escherichia coli* as the cause of moderate-to-severe diarrhea in children <5 years in Bangladesh among other countries (Kotloff et al., 2013). Worldwide, the annual burden of *Shigella* is estimated to be 164.7 million cases, with 163.2 million from developing countries, resulting in 1.1 million deaths, and 69% of which are in children <5 years of age (Kotloff et al., 1999). The exact demography is unclear, however, as a previous study conducted in six Asian countries showed that the burden of shigellosis was 2.1 per 1000 population per year for all ages, and for children under 5 years, it was 13.2 per 1000 population every year (von Seidlein et al., 2006).

*Shigella* is highly infectious because 10 *Shigella* colonies are enough to cause disease (DuPont et al., 1989). There are four species of *Shigella*, based on biochemical and serological properties: *S. dysenteriae*, *S. flexneri*, *S. boydii*, and *S*. *sonnei* (Livio et al., 2014). These species are further classified into 15, 23 (including subtypes), 20, and 1 serotype, respectively (Talukder and Azmi, 2012; Shahnaij et al., 2018). *S. boydii* has been reported less frequently worldwide compared to other *Shigella* species (Bratoeva et al., 1992; Ranjbar et al., 2008) and there are very few published studies available on few serotypes of *S*. *boydii* (Kania et al., 2016). GEMS showed that, over 3 years, 5.4% (61/1130) of all *Shigella* were identified as *S. boydii* (Livio et al., 2014). Although this is a small contribution compared to the other three *Shigella* species, *S. boydii* still makes up a significant component of the overall *Shigella* burden (Baker et al., 2015).

*Shigella* spp. are currently identified by biochemical tests and suspected colonies are confirmed by serotyping (Grimont et al., 2007) using commercially available antisera. Most *Shigella* O-antigens serologically cross-react with O-antigens of some *E. coli* strains (Liu et al., 2008), making identification difficult. Both demonstrate similar biochemical properties and can cause dysentery using the same mechanism (Ud-Din and Wahid, 2014). The 16S rRNA sequence similarities of *S. flexneri*, *S. boydii*, and *S. sonnei* with *E. coli* were reported to be 99.8, 99.7, and 99.9%, respectively (Fukushima et al., 2002). *S. boydii* and *S. dysenteriae* were considered physiologically similar, but differed biochemically by the mannitol test (Muthuirulandi Sethuvel et al., 2017). Although various molecular methods have been proposed in the past years, discriminating between species is still difficult (Pettengill et al., 2015; Muthuirulandi Sethuvel et al., 2017). Recently developed whole-genome sequence (WGS) based methods showed better discrimination between closely related species and provided clinically relevant information (Hasman et al., 2014). The k-mer-based identification approach derived from WGS data effectively differentiated *Shigella* from *E. coli* and accurately provided information on phylogenetic relationship (Chattaway et al., 2017). However, these are no cost-effective methods and those that do exist require specialized training and equipment.

The WHO designated *Shigella* as a priority area for research and development of new drugs (World Health Organization, 2017). To better understand different serotypes of *Shigella*, it is important to type or distinguish them accurately as immunity to *Shigella* is serotype specific (Mani et al., 2016). *Shigella* serotype-specific lytic phages are useful for typing *Shigella* at serotype level and thus constitute a powerful diagnostic tool. Phage typing is a rapid, consistent, and reproducible technique requiring minimal specialized equipment (Anderson and Williams, 1956; Adams, 1959; Ahmed et al., 1995).

The purpose of this work was to show the prevalence of 20 different serotypes of *S. boydii* in Bangladesh and diagnosis of *S. boydii* serotypes without using antisera. In this study, we have isolated phage MK-13 and evaluated its use for diagnosis of *S. boydii* type 1, one of the prevalent serotypes of *S. boydii* in Bangladesh. This is the first report of the isolation and whole genome sequence analysis of a phage that lyse only strains of *S. boydii* type 1 but not other 19 serotypes of *S. boydii* or other species of *Shigella*. So far, no serotype-specific phage has been reported for diagnosis of *S. boydii* type 1.

## Materials and Methods

### Bacterial Strains Analyzed for Serotype Distribution of *S. boydii*

A total of 6475 strains of different serotypes of *Shigella* were isolated and identified at the Enteric and Food Microbiology Laboratory from patients of all ages with diarrhea attending the Dhaka treatment center of the International Centre for Diarrhoeal Disease Research, Bangladesh (icddr,b) hospital and diagnostic unit between 1999 and 2015 following the standard microbiological, biochemical, and serological methods (World Health Organization, 1987; Talukder et al., 2002). Of 6475 strains, 877 were found as *S. boydii*. From 877, 793 strains were serotyped and further analyzed in this study (Supplementary Table S1). It should be mentioned here that all the samples couldn’t be collected in 2014 and 2015, and therefore, the isolation rate is low in these 2 years. All the strains were stored in Tryptic Soy Broth (TSB) with 0.3% yeast extract (TSBY) including 15% glycerol at −80°C in icddr,b bio repository facility.

### Bacterial Strains Used for Host Range Determination of Bacteriophages

A total of 342 strains of clinical *Shigella*, *E. coli*, *Salmonella*, *Vibrio*, *Klebsiella*, *Proteus*, and *Yersinia* of which 294 strains belonged to 48 different serotypes of *Shigella* randomly selected from different years and 48 other enteric bacteria were used for testing phage susceptibility (Table 1 and Supplementary Table S2). These strains were collected from the Enteric and Food Microbiology laboratory of icddr,b, Dhaka, Bangladesh. *S*. *boydii* type 1, K-473 was used as host strain for detection of phage MK-13.

**TABLE 1:** Strains used for testing the susceptibility of MK-13.

	**Name of organisms tested**	**No. of strains tested (*n* = 342)**	**No. of isolates susceptible to MK-13**
*Shigella* (*n* = 294)	*S. flexneri* serotypes 1a, 1b, 1c, 2a, 2b, 3a, 3b, 4a, 5a, 5b, 6a, and 6b, type 4, and variants *X*, *Y*, and *Z*	99	0
	*S. boydii* 1	10	10
	*S. boydii* 2	2	0
	*S. boydii* 3	2	0
	*S. boydii* 4	6	0
	*S. boydii* 5	3	0
	*S. boydii* 7	5	0
	*S. boydii* 8	8	0
	*S. boydii* 9	2	0
	*S. boydii* 10	5	0
	*S. boydii* 11	7	0
	*S. boydii* 12	10	0
	*S. boydii* 13	6	0
	*S. boydii* 14	11	0
	*S. boydii* 15	8	0
	*S. boydii* 16	2	0
	*S. boydii* 18	5	0
	*S. boydii* 19	1	0
	*S. boydii* 20	2	0
	*S. dysenteriae* types 1 to 4, 6, 8, 9, and 11 to 15	63	0
	*S. sonnei*	37	0
*E. coli* (*n* = 29)	ETEC	9	0
	EPEC	8	0
	EAEC	10	0
	EHEC	1	0
	EIEC	1	0
*Salmonella* (*n* = 13)	*S. typhi*	4	0
	*S. paratyphi*	7	0
	Other *Salmonella*	2	0
*Vibrio cholera* (*n* = 3)	01	2	0
	0139	1	0
*Klebsiella* (*n* = 1)	Kp-9	1	0
*Proteus* (*n* = 1)	CF-53	1	0
*Yersinia* (*n* = 1)	Bact U22	1	0

### Isolation and Identification of Enteric Bacteria Used for Phage Host Range Determination

All enteric bacteria were identified and isolated following the standard microbiological and biochemical methods (World Health Organization, 1987). Shortly, stool samples were first sub-cultured on different agar media such as MacConkey agar, Shigella-Salmonella (SS) agar, xylose lysine deoxycholate (XLD) agar, thiosulfate citrate bile salts sucrose (TCBS), taurocholate tellurite gelatin agar (TTGA), and incubated at 37°C overnight for all plates. The suspected colonies were then confirmed by short biochemical tests, oxidase reaction, and serology. Different *E. coli* pathotypes were identified according to the protocol standardized by the MAL-ED study investigators (Houpt et al., 2014). All the isolated strains were stored in TSBY including 15% glycerol at −80°C in icddr,b bio repository facility.

### Serotyping of Different Serotypes of *Shigella*

*Shigella* isolates were serotyped using two kits: a commercially available antisera-kit (Denka Seiken, Tokyo, Japan) and type- and group-specific monoclonal antibody reagents (Reagensia AB, Stockholm, Sweden). Strains were sub-cultured onto MacConkey agar (Difco) plates, and after 16–18 h of incubation, slide agglutination tests were performed following *Shigella flexneri* serotyping scheme as described previously (Talukder and Azmi, 2012). For the other three species, the manufacturer’s guidelines were followed.

### Collection of Water Samples and Enrichment for Detection and Isolation of Phages

Twenty-eight water samples were collected in sterile containers from different rivers, canals, and lakes in and around Dhaka City, Bangladesh, between February 2006 and February 2007 for isolation of *Shigella-*specific phages. Within 3 h of collection, water samples were mixed with equal volume of 2 × Luria-Bertani (LB) broth and overnight culture of *Shigella* (one strain of *S. flexneri* per serotype 2a, 2b, 3a, 1c; *S. boydii* 1, 12; *S. dysenteriae* 1, and *S. sonnei*) and incubated overnight at 37°C. The suspensions were then centrifuged at low speed, carefully transferred to another tube, and filtered through 0.22-μm pore-sized filters (Millipore). Filtered supernatants were then screened for presence of phages. Briefly, 500 μl of logarithmic-phase cells of the host strain in LB broth was mixed with 3.5-ml aliquots of soft agar (LB broth containing 0.8% Bactoagar, Difco), and the mixture was overlaid on previously prepared LB agar plates. Filtered supernatants were then inoculated on the plates and incubated for 16–17 h at 37°C. A sample was counted as positive for phages when plaque was observed on the bacterial lawn (Sambrook et al., 1989).

### Isolation, Propagation, and Purification of the Phage

The samples positive for phages were then serially diluted, mixed with soft agar, and overlaid on hard bottom agar and incubated for 16–17 h at 37°C as described above. Next day, lytic plaque zones were collected by cutting the soft layer from the plate using sterile cut tips and placing them separately in 500 μl of logarithmic-phase cells, vortexed, and incubated at 37°C for 20 min. Then 3 ml of LB broth was added and incubated overnight at 37°C. Then, the culture was centrifuged at 10,000 × *g* for 20 min, and the supernatant was filtered through 0.22-μm pore-sized filters (Millipore) to exclude bacteria. The number of phage particles in the filtered supernatant was determined by testing serial dilutions of the supernatant with the host strain. This method was repeated for three successive times to obtain purified phages.

### Determination of Host Range of the Phages

After purification, the phages were tested using 48 different serotypes of *Shigella* and then the selected phages were tested on other enteric bacteria (Table 1). Briefly, an overnight culture of the host strain was diluted 1:100 in fresh LB broth and grown at 37°C for 4 h. From this, 500 μl was added to 3 ml of LB broth and mixed with 3.5-ml aliquots of soft agar, and the mixture was overlaid on previously prepared LB agar plates. Each overlay was allowed to solidify for 15 min. Ten microliters of each purified phage at a titer of 10^3^ PFU/ml was spotted onto the bacterial overlay, dried, and incubated overnight at 37°C. The tests were repeated three times to confirm the results.

### Electron Microscopy of the Phage Particles

A high-titer phage preparation (∼10^11^ PFU/ml) was obtained using the plate lysis procedure as described previously (Albert et al., 1996). Carbon-coated copper grids were used for morphological studies of bacteriophage. Grids were subjected to glow discharge before negative staining with 2% uranyl acetate. Samples were examined in a FEI Tecnai12BioTwin transmission electron microscope operating at an acceleration voltage of 100 kV and fitted with a SIS Megaview III CCD camera. All measurements were done using analySIS software (Ghosh et al., 2005).

### Isolation and Analysis of Phage Nucleic Acids

Phage nucleic acid was extracted following a previously described method (Faruque et al., 2003). Briefly, the filtrates were mixed with one-fourth volume of a solution containing 20% polyethylene glycol (PEG-6000) and 10% NaCl. Then, the mixture was centrifuged at 13,000 × *g* and the pellet was dissolved in a solution containing 20 mM Tris–HCl (pH 7.5), 60 mM KCl, 10 mM MgCl_2_, and 10 mM NaCl, and digested with DNase I and RNase A at 37°C for 2 h. The solution was then extracted with phenol-chloroform. Ethanol was used to precipitate total nucleic acids, which were suspended in deionized water and purified using the SV Minipreps DNA purification system (Promega, Madison, United States). To initially check for diversity, the isolated phage nucleic acids were digested with *Eco*RI, *Xba*I, *Hin*dIII, *Mlu*I, *Sal*I, and *Hae*III (Invitrogen Corporation, Carlsbad, CA) according to the manufacturer’s recommendations (Sambrook et al., 1989) and analyzed by agarose gel electrophoresis following standard procedures.

### Whole Genome Sequencing of the MK-13 Phage Genome

The phage genome was sequenced at the University of Leicester, United Kingdom, using the Illumina MiSeq platform. The genomic library was prepared using the Illumina Truseq Nano DNA library Preparation Kit as per the manufacturer’s instructions and sequenced with the MiSeq Reagent Kit v3 (600 cycles) to yield 300-bp paired-end short DNA reads.

The quality of the raw sequencing reads was checked using FastQC (Andrews, 2010). The sequencing reads were filtered based on a threshold mean Phred quality score of 20 using skewer (Jiang et al., 2014). Reads were assembled with SPAdes-3.10.1 (Bankevich et al., 2012). Although reads were assembled using multiple k-mer lengths, the longest contig was assembled using k-mers of 127 bp in length and thus used for further analysis. We only considered the longest contig as being the phage genome as the coverage of the other contigs was 5–10 times lower. The phage genome was circularly permuted, resulting in direct repeats at each end. The repeats were identified and trimmed with the apc.pl script written by Joe Fass^1^. Open reading frames were annotated on the phage genome sequence with PROKKA (Seemann, 2014) and then fine-tuned using the RAST annotation server (Aziz et al., 2008). The COG functional classification was performed by submitting the protein sequences to NCBI Batch CD-search Marchler-Bauer et al. (2015). The visualization of genomic organization is made using DNA plotter (Carver et al., 2008).

### Genome Analysis and Comparison

Genomic organization of MK-13 phage was visualized using DNA plotter (Guy et al., 2010; R Core Team, 2013). Also, accessory genome-based phylogeny was built as there was no single gene of MK-13 that is shared among all currently available 35 *Shigella* phage genomes within NCBI (Supplementary Table S3). All the phage genome annotation was done using PROKKA, then using the gff files, core genome analysis was performed using tool Roary (Page et al., 2015) using default parameters. As we are unable to find any single core gene among all the genomes, we adopted the phylogeny based on gene presence–absence matrix. Therefore, we used the phylogeny tree generated by Roary (newick flat file) and visualized in R package “ggtree.” FastTree was used for the phylogeny construction, which infers approximate-maximum-likelihood phylogenetic trees. These accessory genes may or may not be shared between two phages and whether the particular sets of phages have high or low number of shared genes should be a reasonable indicator of their relatedness. The Genbank file of *Shigella* phage ΦSboM-AG3 was downloaded from NCBI (accession number FJ373894.1). The comparison of this phage against MK-13 was performed using EasyFig (Sullivan et al., 2011).

### Data Availability Statement

The MK-13 phage genome is deposited under accession number MK509462 in NCBI Genbank database.

## Results

### Serotype Distribution of *S. boydii*

**FIGURE 1:**
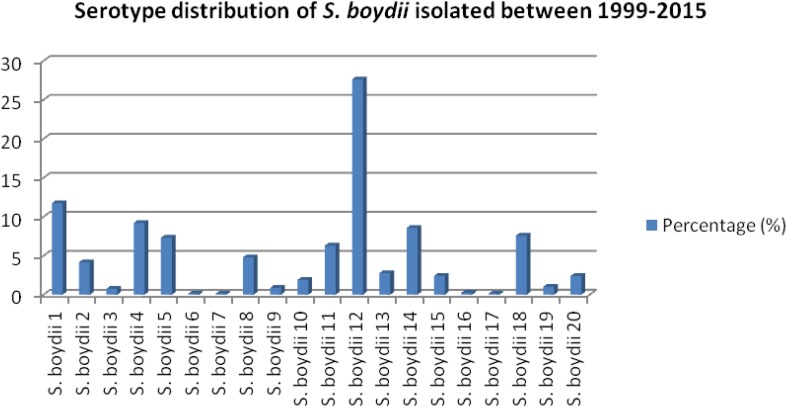
Prevalence of different serotypes of *S. boydii* in Bangladesh.

Among 6475 *Shigella*, 13.54% (877/6475) were identified as *S. boydii*. Among 793 strains of *S. boydii*, *S. boydii* type 12 is the prevalent serotype (27.6%) followed by *S. boydii* type 1 (11.7%), type 4 (9.2%), type 14 (8.6%), type 18 (7.6%), type 5 (7.3%), type 11 (6.3%), type 8 (4.8%), type 2 (4.2%), type 13 (2.8%), types 15 and 20 (2.4% each), and others are below 2% (Figure 1).

### Detection and Isolation of Phages From Environmental Water Samples

From 28 water samples, 84 *Shigella*-specific phages were isolated. Of these, 15 different types of phages were selected for purification. Among these, only one phage designated as MK-13 was found to be specific for *S. boydii* type 1, which was selected for further host range characterization.

### Host Range, Plaque Morphology, and TEM of Phage MK-13

**FIGURE 2:**
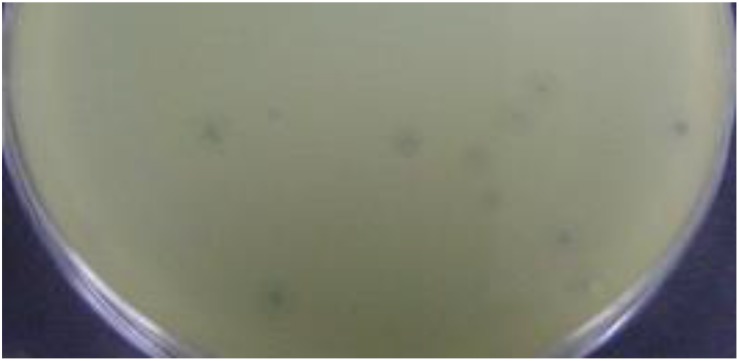
Plaque morphology of MK-13 phage.

The purified MK-13 phage lysed all strains of *S. boydii* type 1 only, but not the other 47 serotypes of *Shigella* or other enteric bacteria tested (Table 1). Phage MK-13 produced completely clear plaques with outer turbid zone and about 3 mm diameter on a lawn of *S. boydii* type 1. When grown in LB broth with control host, the phage produced a titer of 10^9^ PFU/ml. Plaques of MK-13 are slightly bigger than most of the other phages tested (Figure 2).

**FIGURE 3:**
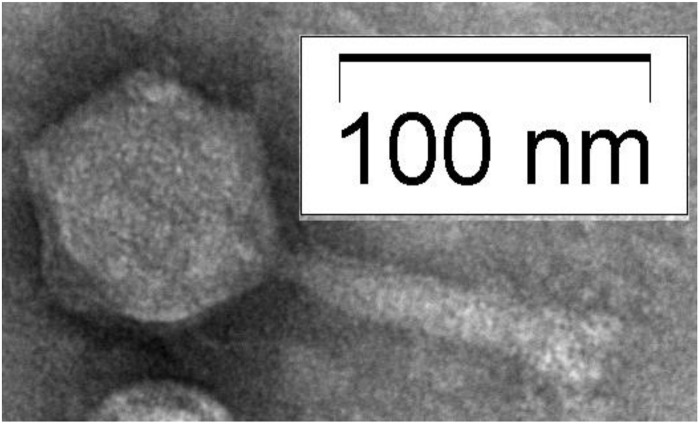
Electron micrograph showing the morphology of *Shigella boydii* type 1 specific phage.

Electron microscopic examination of the phage showed that the phage has an icosahedral head, and a long contractile tail (Figure 3). The head is approximately 85 nm in diameter and the contractile tail is approximately 110 nm long and 15 nm in diameter. Therefore, the phage belongs to *Myoviridae* family.

### Restriction Profiles of the Isolated Phage DNA (RFLP)

**FIGURE 4:**
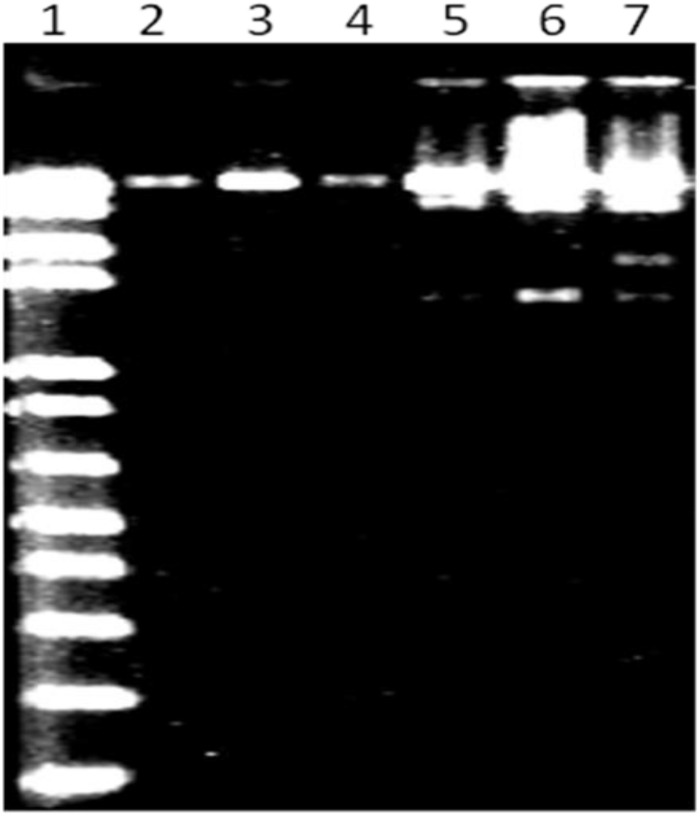
*Mlu*I restriction profile of MK-13 phage DNA (lane 7). Lane 1 shows 1-kb Plus DNA ladder (Invitrogen) and lanes 2 to 6 show some of the other phages isolated in this study.

MK-13 phage DNA was sensitive to *Hae*III, *Mlu*I, and *Hin*dIII restriction enzymes, but resistant to *Eco*RI, *Xba*I, and *Sal*I. This pattern of sensitivity was different from that of DNA from other phages tested. Restriction profile of MK-13 phage DNA is shown in Figure 4.

### Genomic Analysis of MK-13 Phage

**FIGURE 5:**
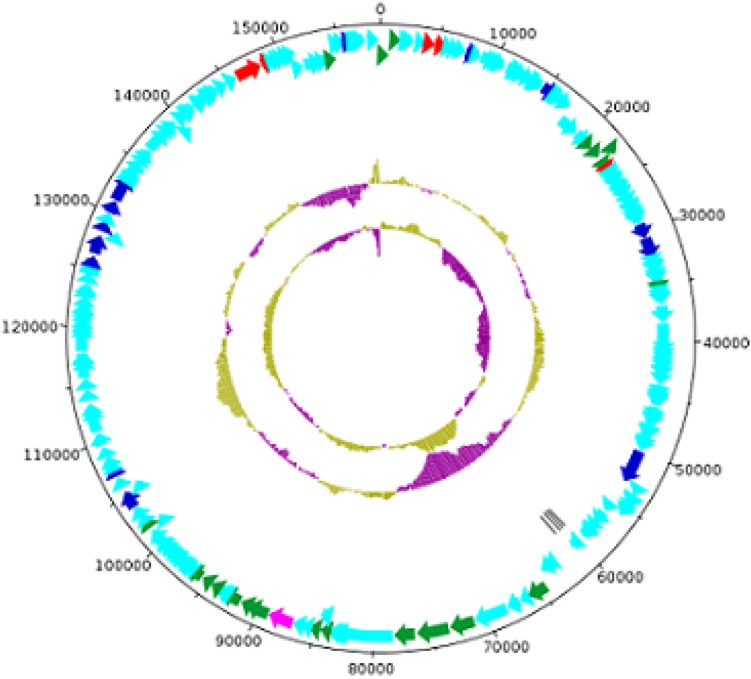
Circles from inside to outside indicate GC skew, GC content, tRNA genes on reverse strand, CDS on reverse strand, and CDS on forward strand. The COG functional categories are represented–blue (L: replication, recombination, and repair), red (F: nucleotide transport and metabolism), and pink (X: prophages and transposons). Structural genes of MK-13 phage are shown in green color.

The MK-13 phage (GenBank accession number: MK509462) contains a double-stranded linear DNA genome composed of 158,755 bp with a G + C content of 49.45%. A total of 211 open reading frames (ORFs) were identified, of which 4 included tRNA coding regions with specificity for asparagine (GTT), isoleucine (GAT), serine (TGA), and tyrosine (GTA). The remaining 207 ORFs encoded putative proteins. For the initial characterization of the phages, the contigs generated were automatically annotated with RAST. The annotated proteins were classified into different groups mainly as DNA replication, recombination, nucleotide metabolism, transcription, translation, phage structure and packaging, lysis, and phage–host interactions. Genes involved in the lysogenic process such as an integrase, repressor, or holin were not recognized in this phage. A list of phage structural proteins and some replication and regulatory proteins are shown in Supplementary Table S4 and a DNA plotter is presented in Figure 5. Annotation of MK-13 phage identified most ORFs as “hypothetical proteins” without putative functions, which is common among highly novel phages.

### Phylogenomics and Gene Synteny

BLAST analysis revealed that the newly sequenced phage MK-13 was distinct from the publicly available phages in databases except for ΦSboM-Ag3. At the time of writing this manuscript, 35 complete genomes of *Shigella* phages were found in public databases. Only two of these phages, P^*Sb–*1^ (Jun et al., 2014) and ΦSboM-Ag3 (Anany et al., 2011), were found to infect *S. boydii*. Phage P^*Sb–*1^ infects ATCC 8700 (*S. boydii* type 2) and ATCC 35966 (*S. boydii* type 18) but not the other species of *Shigella* or other bacteria tested. Whereas phage ΦSboM-AG3 was reported to have a broader host range including most of the *S. boydii*, *S. flexneri*, *S. dysenteriae*, and *S. sonnei* tested. Phylogenetic tree constructed with MK-13 and other *Shigella*-specific phages revealed that MK-13 phage is closely related to phage ΦSboM-AG3. RAST annotation showed that MK-13 had 89% query coverage with 98% sequence identity with ΦSboM-AG3. The phage family, genome length, GC content, tRNAs, CDS, and most of the other properties of phage MK-13 are similar to phage ΦSboM-AG3 but distant from *Shigella* phage P^*Sb–*1^ (Table 2).

**TABLE 2:** Phenotypic and genomic properties of MK-13 compared with bacteriophages ΦSboM-AG3 and P^Sb–1^.

**Phage**	**ΦSboM-AG3**	**MK-13**	**p^Sb–1^**
Host range	*S. boydii*	*S. boydii* type 1	*S. boydii* type 2, *S. boydii* type 18
	*S. flexneri*		
	*S. dysenteriae*		
	*S. sonnei*		
Family	Myoviridae	Myoviridae	Podoviridae
GenBank accession no	FJ373894	MK509462	KF620435
Genome length (bp)	158,006	158,755	71,629
GC content (%)	50.4	49.45	42.74
Overall DNA sequence identity with MK-13 (%)	90% query covered with 98% identity	–	0% query covered
Putative proteins (RAST annotation)	68	73	60
No. of CDS	216	207	103
tRNAs	tRNA-Asn-GTT	tRNA-Asn-GTT	1 tRNA (undetermined)
	tRNA-Tyr-GTA	tRNA-Tyr-GTA	
	tRNA-Ser-TGA	tRNA-Ser-TGA	
	tRNA-Ser-GCT	tRNA-Ile-GAT	

**FIGURE 6:**
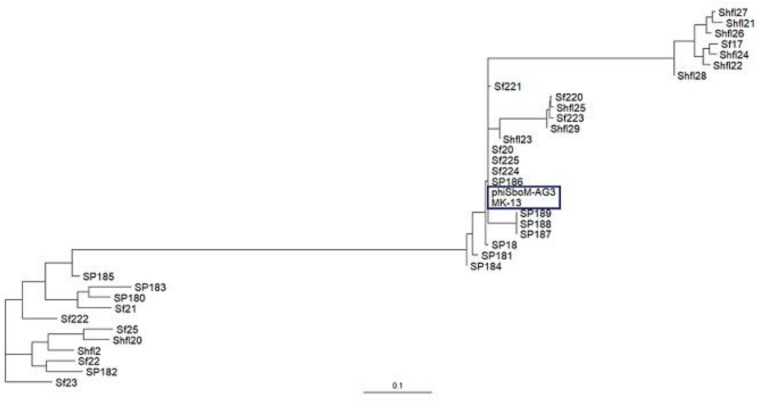
Phylogenetic interrelationship among the selected *Shigella* phages. The dendrogram represents the maximum-likelihood phylogenetic tree, which is based on the gene presence–absence data. The blue color box is used to indicate the selected phages of *Shigella boydii*. The scale bar represents accessory gene content similarity across the branch length.

**FIGURE 7:**
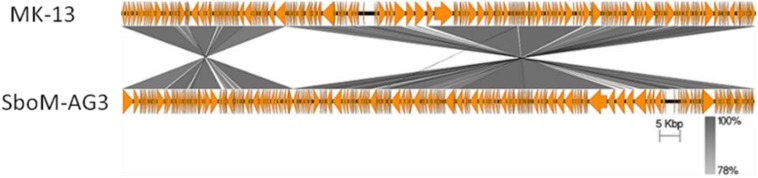
Genomic comparison of phage MK-13 with *S. boydii* phage ΦSboM-AG3. The gray color intensity is proportional to the sequence similarity as indicated in the scale below.

The MK-13 genome displayed two inversions compared to ΦSboM-AG3 genome. The first inversion includes the genomic region of around 41 kb nucleotides of MK-13, whereas it comprised more than 42 kb nucleotides in ΦSboM-AG3. Interestingly, the DNA ligase protein in the first inversion had the most variability among all the core proteins of ΦSboM-AG3 and MK-13. In case of the second and largest inversion, the MK-13 genome was longer (116 kb) than the ΦSboM-AG3 (115 kb). The breakpoints of the genomes were adjacent to the phage rII lysis inhibitor genes. The phylogenetic tree and gene synteny are shown in Figures 6, 7, respectively.

### Gene and tRNA Function Compared Between MK-13 and SboM-AG3

Phage MK-13 genome encodes four tRNA genes, of which three match with ΦSboM-Ag3 phage but did not match with any of the tRNA of P^*Sb–*1^ (Tables 2, 3). Function-based comparison through RAST showed that only two proteins are common among the two phages specific for *S. boydii* (Table 4).

**TABLE 3:** Function based comparison among the 3 phages specific for *S. boydii*.

**Category**	**Unique proteins of MK-13 compared to ΦSboM-AG3**	**Unique proteins of ΦSboM-AG3 compared to MK-13**	**Common proteins among MK-13, ΦSboM-AG3 and P^Sb–1^**
DNA replication	–	–	DNA helicase, phage-associated
Regulatory protein	Putative glutaredoxin	–	–
Structural protein	Phage tail fiber	–	Phage tail fibers
	Single stranded DNA-binding protein	–	–
Others	Phage recombination-related endonuclease Gp46	–	–
Hypothetical protein	Hypothetical protein (*n* = 11)	Hypothetical protein (*n* = 17)	–
tRNA	tRNA-Ile-GAT	tRNA-Ser-GCT	–

**TABLE 4:** Common proteins among MK-13 and P^Sb–1^ with their functions.

**Length (bp)**	**Gene ID (MK-13)**	**Function**	**Gene ID (P^Sb–1^)**	**Identity (%) with (P^Sb–1^)**	**Function**
110	CDS 27	Phage protein	CDS 50	33.68	Phage protein
270	CDS 115	Phage tail fiber	CDS 57	34.09	Phage neck whiskers
521	CDS 117	Phage rIIB lysis inhibitor	CDS 77	28.57	Phage rIIB lysis inhibitor
919	CDS 118	Phage rIIA lysis inhibitor	CDS 76	21.77	Phage rIIA lysis inhibitor
965	CDS 153	Hypothetical protein	CDS 22	41.85	Phage tail fibers
595	CDS 156	Phage tail fibers	CDS 19	54.7	Hypothetical protein
141	CDS 182	Hypothetical protein	CDS 47	27.2	Bll0063 protein

### Sequence-Based Comparison Among MK-13, SboM-AG3, and P^Sb–1^

Sequence-based comparison through RAST among MK-13 and ΦSboM-AG3 phages showed that the main differences among the CDSs are in hypothetical proteins. A total of 11 hypothetical proteins and one phage protein are found unique for MK-13, which did not match with any CDS of ΦSboM-AG3. However, 17 hypothetical proteins of ΦSboM-AG3 are unique compared to MK-13. BLAST alignment of the unique hypothetical proteins of MK-13 is shown in Supplementary Table S5.

Another CDS of MK-13 has been detected, which showed 97% identity with 78% query cover with ΦSboM-AG3 where their functions were different. Function of this sequence in MK-13 is ribose-phosphate pyrophosphokinase (EC 2.7.6.1), which showed closest match to more than 30 phages. However, the function in ΦSboM-AG3 is nicotinamide phosphoribosyltransferase (EC 2.4.2.12), which didn’t show any close match to other phage sequences. Sequence-based comparison among the MK-13 and P^Sb–1^ phages showed seven CDSs from MK-13 matched with P^Sb–1^ with 21–55% identity, of which functions of three CDSs are same (Table 4). Six CDSs of ΦSboM-AG3 matched with CDSs of P^Sb–1^ with 21–53% identity having similar functions. From these six CDSs of ΦSboM-AG3, five also matched with CDSs of MK-13 with similar functions (Table 5). Common proteins among MK_13 and ΦSboM-AG3 are listed in Supplementary Table S6.

**TABLE 5:** Common proteins among ΦSboM-AG3 and P^Sb–1^ having similar functions.

**Length (bp)**	**Gene ID (SboM-AG3)**	**Function**	**Gene ID (PSb-1)**	**Percent identity**
917	CDS 1	Phage rIIA lysis inhibitor	CDS 76	21.77
521	CDS 2	Phage lysis inhibitor # T4-like rIIA-rIIB membrane associated #T4 GC169828.57	CDS 77	28.57
110	CDS 93	Phage protein	CDS 50	33.68
141	CDS 136	Hypothetical protein	CDS 47	25.81
595	CDS 161	Phage tail fibers	CDS 19	52.99
1020	CDS 164	Hypothetical protein	CDS 22	38.36

## Discussion

Of all the enteric bacteria including 20 serotypes of *S. boydii* tested in this study, only *S. boydii* type 1 was susceptible to phage MK-13. Hence, this phage appears to be a useful diagnostic tool (Olsen et al., 1993). Most of the other phages tested infected a broader spectrum of *Shigella* strains belonging to different species or serotypes.

Electron microscopic examination and WGS of the phage revealed that it belonged to the *Myoviridae* family (Hendrix, 2005). Its virion morphology is very similar to phage ΦSboM-AG3 (Anany et al., 2011). The MK-13 and ΦSboM-AG3 genomes shared nucleotide identity and gene synteny, providing evidence that they are closely related. However, only one structural protein, phage tail fiber, is the unique protein that is not found in phage ΦSboM-AG3. This protein may have a role in a narrow host range specificity of MK-13. ΦSboM-AG3 was reported to have a broader host range, where MK-13 showed specificity to its single host. Phage P^Sb–1^ is highly specific to *S. boydii*, but its genome is only distantly related to the MK-13 genome. Breakpoints in synteny between the MK-13 and the ΦSboM-AG3 genomes were adjacent to phage rII lysis inhibitor genes. Mutation of these genes is known to alter the speed of lysis (Dressman and Drake, 1999; Chen and Young, 2016). Mosaicism (breaks in synteny) in phage genomes is a common reported phenomenon and may contribute to phage evolution and adaptation (Hatfull, 2008; Belcaid et al., 2010).

To detect *S. boydii* type 1 in an unknown sample using the current standard antisera approach, a set of polyvalent and another set of monovalent antisera are necessary for a series of agglutination tests to confirm a strain as *S. boydii* type 1 whereas 2 μl at 10^6^ titer of MK-13 phage can alternatively be used for detection of *S. boydii* type 1, which is faster and cheaper. The production, purchasing, and shipment of antisera from the producer company are costly and time consuming. In contrast, phage production is easier, cheaper, and efficient. Developing a *Shigella* phage typing scheme will improve the speed, efficiency, and accuracy instead of the current standard *Shigella* serotyping scheme. Phage typing could provide better results than serotyping and have greater discriminative power compared to other commonly used methods. Phage MK-13 will be useful to confirm the diagnosis of infection caused by *S. boydii* type 1 and for differentiating it from other *Shigella* serotypes, and *Shigella* like-organisms, *E. coli*, and other non-lactose fermenting colonies.

Several typing techniques are used to identify different bacteria but the cost compared to phage typing is prohibitive. Though serotyping is the most prevalent effective diagnostic in resource-poor settings, phage typing has been proven as a rapid and low-cost approach for efficient diagnosis, epidemiological surveillance, and outbreak investigation but needs trained and experienced technicians. The technique has been adopted successfully within certain contexts in microbiology, such as *Staphylococcus* outbreaks in hospitals and nurseries in the 1950s (Hillier, 2006). In another study, for detection of *Vibrio parahaemolyticus*, the authors combined the technique with a coupled enzyme system *in vitro* because of its rapid detection, high specificity, and simplicity in operation (Peng et al., 2014).

The limitations of our study were the host specificity of MK-13 was not tested for *S. dysenteriae* types 5, 7, and 10; *S. flexneri* 4b; and *S. boydii* type 6 and 17, which were not available in the lab. These serotypes are not prevalent in Bangladesh. The precise bacterial epitope to which phage MK-13 binds is unknown, and it is not clear whether this epitope is encoded in a non-*S. boydii* type 1 strain that was not tested in this study.

Previously described *Shigella* phages infected a broader spectrum of *Shigella* belonging to different species or serotypes (Goodridge, 2013; Jun et al., 2016; Hamdi et al., 2017; Soffer et al., 2017). This study is the first one to address *Shigella* phage for typing of *Shigella* for diagnosis of shigellosis at the serotype level. The phage, MK-13, isolated in this study can be used as a cheap and reliable diagnostic marker for detection of shigellosis caused by *S. boydii* type 1 as an easier, cheaper, and efficient method in less developed countries, especially in rural areas. Although not the focus of this study, the phage may also have potential to remove bacterial contamination in water and foods that are not cooked before eaten, and in epidemiological application and treating. Further work will focus on testing more strains of different *S. boydii* serotypes and to identify the precise bacterial epitope to which phage MK-13 binds.

## Data Availability Statement

The datasets generated for this study can be found in the MK509462.

## Ethics Statement

Experimental protocol was approved by the Research Review Committee (RRC) and the Ethics Review Committee (ERC) of the icddr,b (Protocol Number PR-14040). All methods were conducted in accordance with the guidelines of the RRC and ERC.

## Author Contributions

MA: conception, study design, laboratory experiments, genomic data analysis, writing of the original manuscript, editing, finalizing, and took overall responsibilities of the project as principal investigator. NB: library preparation, whole genome sequencing, genomic data assembly, review of annotation, and editing of the manuscript. MC: whole genome sequencing and review, and editing of the manuscript. MY: laboratory experiments. TT: genomic data analysis. RB: review of genomic data analysis and editing of the manuscript. MAA: laboratory experiments. AG: all preparation and operation for the TEM. NA: planning, supervision, facility for genomic data analysis, and review and editing of the manuscript. KT: conception, study design, manuscript writing and editing, and provided the laboratory facilities to carry out this study. All authors read and approved the final manuscript.

## Conflict of Interest

The authors declare that the research was conducted in the absence of any commercial or financial relationships that could be construed as a potential conflict of interest.

## Footnotes

1 https://github.com/jfass/apc/blob/master/apc.pl
