# Supplementary material for: Prevalence of Shigella boydii in Bangladesh: Isolation and Characterization of a Rare Phage MK-13 That Can Robustly Identify Shigellosis Caused by Shigella boydii Type 1
Source: Front Microbiol. 2019 Nov 7;10:2461. doi: 10.3389/fmicb.2019.02461 (PMC6853846; doi:10.3389/fmicb.2019.02461)
Supplement: Supplementary file 4 [file Data_Sheet_1.doc]

**Supplementary information**

**Prevalence of *Shigella boydii* in Bangladesh: Isolation and characterization of a rare phage MK-13 that can robustly identify shigellosis caused by *Shigella boydii* type 1**

**Tables S4-S5**

**Supplementary Table 4. Structural proteins o**f phage MK-13 including uncategorized proteins.

| **Category** | **gene** |
| --- | --- |
| Structural protein | gp18 tail sheath protein (n=2) |
|  | gp19 tail tube monomer |
|  | Gp23 major head protein |
|  | Gp26 baseplate hub subunit |
|  | gp48 baseplate tail tube cap |
|  | gp6 baseplate wedge subunit |
|  | Membrane-flanked domain |
|  | Phage DNA end protector during packaging |
|  | Phage head completion protein |
|  | Phage neck protein Gp13 |
|  | Phage neck protein Gp14 |
|  | Phage portal vertex |
|  | Phage prohead assembly (scaffolding) protein |
|  | Phage prohead core scaffold protein and protease T4-like phage gp21 T4 GC0129 |
|  | Phage tail completion protein |
|  | Phage tail fiber (n=3) |
|  | Phage tail tube monomer T4-like phage Gp19 |
|  | Putative Gp25 baseplate wedge subunit |
|  | T4-like Phage baseplate wedge component Gp53 |
|  | Tailspike protein |
| Others | Phage virulence-associated VriC protein (n=3) |
|  | Phage rIIA lysis inhibitor |
|  | Phage rIIB lysis inhibitor |
|  | Putative phage-encoded peptidoglycan binding protein |

**Supplementary Table 5. Unique proteins of MK-13 that didn’t match to SboM-AG3.**

| **Length**  **(bp)** | **Gene id** | **Function** | **Closest match to sequences of other phages** |
| --- | --- | --- | --- |
| **103** | CDS 26 | hypothetical protein | 100% query covered with some *E. coli* and *Salmonella* phages with 84-90% identity. |
| **124** | CDS 31 | hypothetical protein | Query covered (100%) only with *Serratia* phage phi MAM1 with 53% identity. |
| **202** | CDS 35 | hypothetical protein | Query covered (100%) only with *Escherichia* phage FEC14, *Salmonella* phage S117 with 99 and 81% respectively. |
| **59** | CDS 38 | hypothetical protein | 91% query covered with *Klebsiella* phages May and Kp110 and *Salmonella* phage SalM PM10 with 74-77% identity. |
| **49** | CDS 43 | hypothetical protein | 100% query covered with 3 *Salmonella* phages SJ2, PM10, 38 with 54-96% identity. |
| **60** | CDS 49 | hypothetical protein | 98% query covered with *Salmonella* phage PM10 with 50% identity. |
| **80** | CDS 50 | hypothetical protein | Query covered with hypothetical proteins of Pectobacterium (97%), Cronobacter (98%), Salmonella phages (63-64%) vB_SalM_PM10, Det7, ViI with (50-60)% identity. |
| **72** | CDS 88 | hypothetical protein | 60-100% query covered with some *Salmonella* and *Dickeya* phages with 77-100% identity. |
| **66** | CDS 141 | hypothetical protein | Query covered (83%) only with *Salmonella* phage SKML-39 with 100% identity. |
| **189** | CDS 142 | hypothetical protein | Query covered (91-100%) with diverse phages including *Salmonella* and *E. coli* phages with 63-100% identity. |
| **173** | CDS 147 | Phage protein | Query covered (85-100%) with more than 90 phages with 42-97% identity. |
| **89** | CDS 179 | hypothetical protein | Query covered only with 2 phages *Citrobacter* phage (100%) and *Escherichia* phage ECML-4 (84%) with 93% and 45% respectively. |
